# The use of and obstacles to social learning in climate change adaptation initiatives in South Africa

**DOI:** 10.4102/jamba.v9i1.292

**Published:** 2017-03-28

**Authors:** Shakespear Mudombi, Christo Fabricius, Verena van Zyl-Bulitta, Anthony Patt

**Affiliations:** 1 Faculty of Management Sciences, Tshwane University of Technology, South Africa; 2 Sustainability Research Unit, Nelson Mandela Metropolitan University, George Campus, South Africa; 3 Faculty of Mathematics and Computer Science, University of Leipzig, Germany; 4 Department of Environmental Systems Science, Swiss Federal Institute of Technology, Switzerland

## Abstract

Global environmental change will have major impacts on ecosystems and human livelihoods while challenging the adaptive capacity of individuals and communities. Social learning, an ongoing adaptive process of knowledge generation, reflection and synthesis, may enhance people’s awareness about climate change and its impacts, with positive outcomes for their adaptive capacity. The objectives of this study were to assess the prevalence of factors promoting social learning in climate change adaptation initiatives in South Africa. An online survey was used to obtain the views of decision makers in government and non-governmental organisations about the presence of personal factors and organisational factors that promote social learning. Descriptive analysis was used to assess these issues. The findings provide some evidence of social learning in climate change adaptation projects in South Africa, with the majority of respondents indicating that personal social learning indicators were present. Mechanisms for improved conflict resolution were, however, less prevalent. The organisational and governance-related barriers to implementation also presented significant challenges. Some of the main organisational barriers were short timeframes for implementing projects, inadequate financial resources, political interference, shortcomings in governance systems and lack of knowledge and expertise in organisations. There is a need for organisations to promote social learning by ensuring that their organisational environment and governance structures are conducive for their employees to embrace social learning. This will help contribute to the overall success of climate change adaptation initiatives.

## Introduction

Climate variability and change are having marked impacts on some communities, with models predicting an increase in the intensity and severity of impacts as well as an increase in the number of people and communities being negatively affected (Adger et al. 2011:757). There is a need to respond to this challenge. As noted, climate change requires urgent action as the costs of early action will be far less than the costs of delay and inaction (Department of Environmental Affairs [DEA] 2011a:10). Dealing with this challenge requires rapid anticipatory responses that empower and enable individuals, communities and decision makers to better adapt to the risks associated with climate change. Adaptation is a process, action or outcome in a system (household, community, group, sector, region or country) that helps the system to better cope with, manage or adjust to some changing condition, stress, hazard, risk or opportunity (Smit & Wandel 2006:282). The goals of adaptation are to moderate the adverse effects of climate change through a wide range of actions targeted at moderating harmful effects while exploiting new opportunities brought about by climate change (Füssel & Klein 2006:304; Intergovernmental Panel on Climate Change [IPCC] 2007:6).

Climate change adaptation challenges are complex, and the trajectories of the systems themselves, as well as human behaviour, are unpredictable. Such challenges have been viewed as ‘messy problems’, as there are large differences between stakeholders regarding the perceptions of the nature of the problem, the need for action and the type of action that should be taken (Pahl-Wostl 2006). Climate change represents a classic multi-scale global change problem characterised by infinitely diverse actors, multiple stressors and multiple time scales (Adger 2006:273). Because of uncertainty, planning for adaptation responses is challenging, requiring that adaptation objectives be set as moving targets that allow for flexibility in implementing responses over time (DEA 2011b:6). Dealing with the challenge requires participation of different stakeholders, Steyaert et al. (2007:540) note that when diverse people are involved in problem-finding and problem-solving processes, they reveal existent but mostly unknown interdependencies, essentially between natural, technical and social phenomena. Different stakeholders understand and perceive the issues differently. So in order for the different actors to work towards the same goal, they have to have a common understanding of the challenge or at least some converging perceptions.

Adaptation is increasingly becoming an important part of policy discussions on climate change; the body of scholarship and the applied work are expanding (Plummer & Baird 2013:635). In addition, social learning is progressively acknowledged as important in enhancing the adaptive capacity (e.g. Allan & Wilson 2009:389; Pelling & High 2005:1). Yet few of these documents and processes explain how adaptive capacity will be enhanced through learning, reflection and innovation. Analysts further do not distinguish between personal factors, at the level of the individual, and organisational factors. The objectives of this study were, firstly, to seek evidence of personal factors, and organisational factors, that promote social learning in climate change adaptation initiatives in South Africa and, secondly, to identify the personal and organisational enablers and obstacles to social learning in these initiatives.

### Background on South Africa

South Africa has an area of about 1 221 000 km^2^ and is located in the southernmost part of the African continent (DEA 2011c:2). The country has nine provinces. In 2015, South Africa had an estimated population of about 54.96 million people (Stats SA 2015). The country has, on average, a semi-arid and warm climate; the prevalent aridity makes the country prone to limitations in water supply (DEA 2011b:ix). South Africa is vulnerable to the negative impacts of climate change, including impacts of increasing temperature and changes in rainfall patterns (both increases in the intensity of rainfall as well as the frequency of droughts) (DEA 2011b:7). Moreover, the relatively high poverty levels compound the vulnerability. The DEA (2011c:x) notes that ‘at least 30% of South Africa’s population is highly vulnerable to both sudden and harmful climatic shocks, with low levels of endogenous resilience, adaptation, and coping skills’.

The South African government has been active in terms of responding to climate change. As set out in the National Climate Change Response White Paper, the country’s vision seeks ‘an effective climate change response and the long-term, just transition to a climate-resilient and lower-carbon economy and society’ (DEA 2011a:5). In relation to adaptation, the objective is to ‘effectively manage inevitable climate change impacts through interventions that build and sustain South Africa’s social, economic and environmental resilience and emergency response capacity’ (DEA 2011a:5).

### Literature review

Climate change requires reflection on successes and failures, and integration of knowledge from several disciplines and sectors (Orderud & Winsvold 2012:956; Shaw & Kristjanson 2013:22). In this context, dynamic learning is more appropriate than conventional blueprint planning in climate change adaptation (Sterman 1994:292). Enhancing the flow of information and development of knowledge and awareness on adaptation is important (Füssel & Klein 2006:321). This requires ongoing, adaptive knowledge creation that is scaled up from individuals though social interactions (McCarthy et al. 2011). This is social learning, viewed by Steyaert et al. (2007:540) as an iterative process of knowledge co-production among interacting stakeholders, resulting in relational and cognitive changes and improved capacities and competencies of actors (Muro & Jeffrey 2012). Thus, social learning is central to climate change adaptation (Collins & Ison 2009:370) by inspiring collective action (Nisbet et al. 2010:329). Multiple stakeholders with different values, beliefs and cultures participate in decision making and action (Blackmore 2007:514). This participation results in the understanding of issues going beyond the individual to become situated within wider social units or communities (Reed et al. 2010).

Social learning is associated with transformations that can be broadly grouped as cognitive, relational, and technical (skills) (Muro & Jeffrey 2012). These transformations are important for climate change adaptation. Cognitive transformation is important because the way people understand and perceive climate change influences their willingness to act and their response to the challenge. Relational transformation is important because climate change has both micro and macro effects, the responses to which require efforts and cooperation by different actors towards a common goal. Technical transformation is associated with improvement in skills, knowledge and the technologies that define the range of possible adaptation responses that individuals and communities can undertake.

A social learning perspective is, however, no panacea to climate change adaptation. Pahl-Wostl (2006) points out that its meaning is very broad, whereas Harvey et al. (2013:12) lament the lack of clarity over definitions and framings of social learning. Some scholars (e.g. Reed et al. 2010; Rodela, Cundill & Wals 2012:16) have commented that some definitions are so broad they could encompass almost any social process, with the same term often being used to refer to different processes (Lotz-Sisitka 2012:11). Moreover, it is not always clear how the social learning theory translates to practice (Allan & Wilson 2009:389) and what the obstacles to social learning are. Impediments to learning may reside in personal cognitive factors which may affect the motivation and capacity of individuals (Mathevet et al. 2011; Sterman 1994:307) or in organisational factors that may frustrate individuals and stifle learning (Stirzaker, Roux & Biggs 2011:4). Social learning is promoted when learning individuals and learning organisations come together. It is therefore important to understand whether efforts to promote social learning should be focused at the organisational level, the individual level, or both.

## Methods

The factors promoting social learning were identified from the literature. These were split into two categories (Table 1). Factors in the *personal social learning* category characterise social learning factors related to the cognitive perceptions and abilities of individuals, whereas those in the *organisational social learning category* relate to organisational characteristics and policies likely to create an environment that facilitates social learning.

**TABLE 1:** Social learning variables used.

Category	Variables	Key references
Personal social learning indicators	Willingness to share ideas with other stakeholders	Pelling and High (2005); Collins and Ison (2009); Nilsson and Swartling (2009); Cundill (2010); Roux et al. (2011); Murray, Roux, and Hill (2010); Muro and Jeffrey (2012); Bos, Brown, and Farrelly (2013); Cundill et al. (2013).
	Development of trust among stakeholders	Pelling and High (2005); Pelling et al. (2008); Collins and Ison (2009); Nilsson and Swartling (2009); Cundill (2010); Roux et al. (2011); Murray et al. (2010); Muro and Jeffrey (2012); Cundill et al. (2013)
	Participation by all stakeholders	Pelling et al. (2008); Cundill and Fabricius (2009); Collins and Ison (2009); Nilsson and Swartling (2009); Cundill (2010); Roux et al. (2011); Murray et al. (2010); Muro and Jeffrey (2012)
	Capacity for conflict resolution	Nilsson and Swartling (2009); Cundill (2010); Roux et al. (2011); Muro and Jeffrey (2012)
	Collective action towards project goals	Pelling and High (2005); Pelling et al. (2008); Collins and Ison (2009); Cundill (2010); Roux et al. (2011); Muro and Jeffrey (2012); Bos et al. (2013)
	Continuous interaction and feedback	Cundill and Fabricius (2009); Collins and Ison (2009); Nilsson and Swartling (2009); Cundill (2010); Roux et al. (2011); Murray et al. (2010)
	Flexibility in planning and implementation	Pelling and High (2005); Pelling et al. (2008); Cundill (2010); Roux et al. (2011); Muro and Jeffrey (2012)
	Willingness to share information	Collins and Ison (2009); Nilsson and Swartling (2009); Cundill (2010); Roux et al. (2011); Muro and Jeffrey (2012); Bos et al. (2013)
Organisational variables	Empowerment of junior employees to experiment	Pelling and High (2005); Pelling et al. (2008); Nilsson and Swartling (2009); Cundill (2010); Bos et al. (2013)
	Existence of processes to translate feedback into changed practices	Pelling et al. (2008); Cundill (2010); Roux et al. (2011); Murray et al. (2010); Muro and Jeffrey (2012); Bos et al. (2013); Tàbara et al. (2010)
	Sufficient budget to regularly engage with stakeholders	Cundill (2010); Roux et al. (2011); Murray et al. (2010); Cundill et al. (2013)
	Continuous updating of project or planning processes	Nilsson and Swartling (2009); Muro and Jeffrey (2012); Tàbara et al. (2010).
	Support for locally initiated projects	Bos et al. (2013); Cundill et al. (2013)
	Development of local stakeholders’ capacity to engage with projects or interventions	Cundill and Fabricius (2009); Collins and Ison (2009); Nilsson and Swartling (2009); Cundill (2010); Roux et al. (2011); Muro and Jeffrey (2012)

Note: Please see the full reference list of the article, Mudombi, S., Fabricius, C., Van Zyl-Bulitta, V. & Patt, A., 2017, ‘The use of and obstacles to social learning in climate change adaptation initiatives in South Africa’, *Jàmbá: Journal of Disaster Risk Studies* 9(1), a292. https://doi.org/10.4102/jamba.v9i1.292, for more information.

Personal social learning indicators referred to factors that individuals had control over, whereas organisational learning indicators were those beyond the control of individuals and which characterised the organisations they worked for.

These variables were used to formulate a questionnaire which was administered through an online survey (https://www.surveymonkey.com/s/RJ5BM2W) in 2013. An online survey method was used because of its advantages compared to other methods, and also given the need to reach many key informants spread all over the country. Evans and Mathur (2005) outlined some of the strengths and weaknesses of online surveys. The strengths include: global reach; flexibility; speed and timeliness; convenience; ease of data entry and analysis; low administration costs; ease of follow-up; and ease to do large samples. The weaknesses include: possible perception as junk mail; skewed attributes of Internet population; challenges with sample selection and implementation; lack of online experience or expertise; technological variation; unclear answering instructions; impersonal; privacy issues; and low response rate. Measures that were used to reduce these likely weaknesses of online surveys, as suggested by Evans and Mathur (2005) included: using an opt-in survey option; brief email with a URL link; simple instruction and easy to answer; use of standard colours and screen dimensions; adequate pre-testing; including respondent names, clarity, and having a highly visible respondent-friendly policy. In addition, follow-up phone calls and sending out reminder emails were used to improve the response rate of the survey.

Individuals working on climate change adaptation and vulnerability reduction initiatives in local, provincial, and national government departments and non-governmental organisations (NGOs) in South Africa were identified. As a first step, a small initial group of key informants were identified through contact lists from climate change conferences and workshops, South African members of climate change online networks such as WeAdapt (http://weadapt.org/) and AfricaAdapt (http://www.africa-adapt.net/) and our personal networks. Thereafter, snowball sampling (Du Plooy 2009:124) was used to increase the number of respondents. Invitations were sent out to 279 individuals of which 38 (14%) completed the survey.

Respondents were asked to indicate whether the personal and organisational social learning factors listed in Table 1 were always, frequently, rarely or never present. They were also asked to rank the list of factors in order of importance, and in an open-ended way, to indicate what the major obstacles to social learning were. The data analysis was performed using Excel and is mainly descriptive.

### Trustworthiness

Steps were taken to ensure reliability and validity in the study. In order to ensure reliability of the collected data, the online questionnaire was evaluated by other experts in the field on whether it measured the right parameters. It was also pilot tested to ensure that it was simple to complete, that the questions were well structured and the coding was proper. The Cronbach’s alpha (Muro & Jeffrey 2012) was used to test the reliability of the parameters used to evaluate social learning in the study. The Cronbach’s alpha seeks to measure the extent to which the scale measures one underlying construct (Field 2009:675); in this case, the construct being measured is ‘social learning’. The scale variables had a Cronbach’s alpha coefficient of 0.808. Pallant (2011) noted that coefficient values above 0.7 are considered acceptable; however, values above 0.8 are preferable. While the sample might appear small, it is important to stress that at the time of the data collection, there were relatively few people working on climate change issues, which might have implications on the generalisation of the results. As pointed out before, the survey was sent out to 279 individuals, of which 38 (14%) completed the survey. The majority of the 38 respondents had significant work experience on climate change issues. About 45% of them had more than 5 years of working experience. About 25% worked for the government (i.e. national, provincial and local), 37% in NGOs, 11% in private organisations, and 29% in research organisations.

## Ethical considerations

Effort was made to ensure that the study was carried out within internationally recognised ethical guidelines. In conducting the study, respondents were not subjected to physical and psychological dangers. They were assured that there was no harm associated with participating in the survey. In addition, the design of the questionnaire ensured that the questions were not offending and discriminatory. Informed consent was sought through invitation phone calls and emails, informing the objectives of the study. Respondents were further informed that participation was voluntary and that they could withdraw from the survey at any time if they felt disinterested as they completed the survey. Furthermore, there was assurance that the data would be used solely for academic purposes and would be kept secret.

## Results

### Presence of social learning variables in adaptation initiatives

The majority of respondents indicated that most of the personal factors promoting social learning were present in adaptation initiatives (Table 2). A four-point Likert scale (never, rarely, frequently and always) was used to understand how the respondents perceived the presence of social learning variables. In this table, to present a clearer picture of the presence of the personal factors that promoted social learning, the percentages of the ‘frequently’ and ‘always’ categories were added (last column). The majority of respondents (> 80%) believed that sharing of ideas with stakeholders, collective action, sharing of information, continuous interaction and feedback, and development of trust were frequently or always present. Other prevalent factors included personal flexibility in planning and implementation of projects and openness to participation by all stakeholders. However, capacity for conflict resolution was less evident, and only 50% of respondents believed it was always or frequently present.

**TABLE 2:** Presence of personal factors that promoted social learning.

Variable	Percentage responses		
	Never (%)	Rarely (%)	Frequently or always (%)
Sharing of ideas with stakeholders	0	0	100
Collective action towards project goals	0	3	97
Participants value of sharing information	0	5	95
Development of trust	0	8	92
Continuous interaction and feedback	0	8	92
Flexible planning and implementation	0	13	87
Participation by all stakeholders	3	11	86
Improved conflict resolution	5	45	50

### Presence of organisational characteristics likely to contribute to social learning

In contrast to personal learning factors, fewer participants believed organisational factors that promoted social learning were present. Sixty-one per cent of respondents indicated that the enabling of stakeholders’ capacity and promotion of locally initiated projects were always or frequently present (Table 3). Factors such as continuous updating of planning processes and empowerment of junior employees were regarded as being frequently or always present by only 39% and 37% of respondents, respectively, while factors such as acceptance of feedback and adequacy of budgets were regarded as being frequently or always present by only 21% and 18% of respondents, respectively.

**TABLE 3:** Presence of organisational factors that contributed to learning.

Variable	Percentage responses		
	Never	Rarely	Frequently or always
The capacity of the local stakeholders to engage with the project is enabled	0	39	61
Most projects are initiated locally	7	32	61
Project or planning processes are continuously updated	5	55	39
Empowers junior employees to experiment	16	47	37
Has processes in place to translate feedback into changed practices	13	63	21
Has sufficient budget to engage with stakeholders repetitively	21	61	18

Respondents ranked capacitation of local stakeholders and promotion of locally initiated projects as the most important in promoting social learning. In addition, respondents suggested two additional factors that contributed to social learning, namely building local-level champions and long-term cooperation with communities. This emphasised the importance of local stakeholders’ participation and empowerment.

### Impediments to learning

When respondents were asked about the factors that seriously undermined the development of a learning environment, their comments^1^ were broadly categorised into six challenges as follows: inadequate funding, short project time frames, political constraints, lack of awareness and knowledge in local communities, lack of trust and organisational governance shortcomings. All of these generally relate to policies and strategies of organisations rather than to personal learning factors.

#### Inadequate funding

Some respondents indicated that funds were simply unavailable, which limited the engagement process, and that rules and conditions imposed by funding agencies and donors were not flexible enough to allow implementers to adjust their work plans. It was also highlighted that the social learning approach is relatively new, and that this presented a constraint in obtaining funding for projects with a learning focus:

- ‘Having enough funds for community engagement is a constant struggle – this is not a traditional “research” area, and so motivating for funds for this kind of work can be hard’.
- ‘Short term nature of funding, which is project rather than programme focused’.
- ‘It is very hard to start initiatives due to lack of funding’.
- ‘The lack of coordination between national, provincial and local governments, and the challenge that rural municipalities typically have the smallest budgets to respond to various community needs’.

#### Short project time frames

This factor is closely related to inadequate funding. Some respondents mentioned that project funding cycles come to an end before an appropriate level of understanding and learning by all stakeholders had been attained. Funds were provided for too short a period to engage all stakeholders and ensure the success of the learning processes:

- ‘Funding limits, not enough time for engagement in some cases, sometimes oversensitivity to partner agendas’.
- ‘Projects/funds come and go, often for short term periods, this strongly undermines long-term engagement with stakeholders, hence undermining the building of trust, communication, reciprocal learning’.
- ‘Unilateral action, and acting too soon before an appropriate level of understanding of the challenges and opportunities is obtained’.

#### Political constraints

Several respondents mentioned that it was difficult to obtain political support for some projects. Others mentioned that political interference affected the attainment of project goals because politicians, especially those at the local level, wanted to seize control of adaptation projects for their own benefit, for example by identifying project beneficiaries from their own constituencies:

- ‘To get political buy-in is very difficult’.
- ‘External influences by those who would benefit from the projects not being implemented’.
- ‘International and national regulations that are strict and sometimes crippling’.

#### Lack of awareness and knowledge in local communities

The very low literacy and education levels in some communities make it difficult to convey awareness raising messages. In addition, project implementers might not actually be able or willing to be involved in the co-production of knowledge. This failure to co-produce knowledge is further worsened by poor communication and feedback. At times, the adoption of the social learning approach is not embraced by the local organisational culture. This presents challenges to the sustainability of projects, particularly when key individuals at the forefront of social learning leave the organisation:

- ‘The current language around climate change is academic and elitist and has not been made accessible to traditional communities and rural audiences’.
- ‘Different levels of education and awareness (related specifically to climate change and adaptation as well as cross-sectoral climate change impacts) among multiple stakeholders can be quite challenging for knowledge sharing. This requires a high adaptive capacity and sophisticated creative thinking from project facilitators and implementers’.
- ‘Project implementers that are not willing to be involved in the co-production of knowledge’.
- ‘The understanding of the importance of the issue within my organisation, is limited to individuals and not always driven by the organisation’.

#### Lack of trust

This was mainly because of differing objectives or goals of various stakeholders, which make it difficult for them to work harmoniously. In addition, some organisations are perceived as dominating, which limits the willingness of other stakeholders to cooperate:

- ‘Lack of trust between stakeholder groups’.
- ‘The differing objectives of donors, practitioners and researchers working together in projects can be difficult to reconcile and threaten the engagement and trust’.
- ‘Some politicians at the local level want to highjack environmental issues for own benefit’.

#### Organisational governance shortcomings

The way implementing organisations are structured, governed and operate represents a significant obstacle. Lack of cooperation and coordination between departments within the same organisation, or between different departments and levels of government (national, provincial and local), was often cited as obstacles to implementation. The slow responses and procurement processes of government hampered timely execution and implementation of projects. In the same context, inappropriate performance evaluation criteria and methods affected project implementation and achievements. For example, organisational performance criteria based on expenditure rather than real impact resulted in a focus on timeous spending of project budgets rather than on enhancing climate change adaptation:

- ‘Ineffective exit strategies and clients – especially government departments whose performance is measured by expenditure rather than real impact’.
- ‘Procurement processes of government institutions; slow response and poor support from national government’.
- ‘Our organisation is perceived as a top down and dominating organisation. Other departments within our organisation are unwilling to open up to new projects’.
- ‘Perception of a private organisation as just being there to make profits’.

## Discussion

Professionals involved in climate change adaptation projects believed that most indicators of personal social learning were frequently present in their projects. The exception, lack of conflict resolution mechanisms, was mentioned by many respondents, and this was believed to be because of conflicting goals (cf. Nilsson & Swartling 2009:4). Some respondents pointed out that project beneficiaries sometimes mistrusted implementation agents, especially if they were private consultants who are perceived mostly as having only a profit motive. The challenges associated with conflict resolution were believed to be exacerbated by lack of consultation by powerful stakeholders who take unilateral decisions. This was also observed by Cundill (2010:10) who found that distrust within a community and between the community and outside agencies affected stakeholders’ ability to translate ideas into shared action.

There were, however, numerous organisational barriers to social learning and most of these related to institutional capacity and governance. Significant obstacles to the implementation of social learning included funding shortages and shortcomings in awareness and knowledge, as well as governance challenges such as political interference, conflict, lack of trust, lack of transparency, and inadequate organisational structures and processes. These are not, however, unique to South Africa. In the Netherlands, Van Bommel, Aarts and Turnhout (2009:410) concluded that the goals, means and methods used by organisations were incompatible with social learning. They further noted that, because of unequal power relations between stakeholders, powerful actors ended up imposing their views on the project. Harvey et al. (2013:35) observed that unequal power and uneven ability to influence were common challenges to the social learning process. Different levels of education and awareness (specifically about climate change adaptation as well as cross-sectoral impacts) among stakeholders can be challenging for knowledge sharing. These lead to ‘powerless spectator communities’ (Fabricius et al. 2007:5) who are either unaware of the threats facing them or have a misguided awareness because of their lack of knowledge. One of the key challenges mentioned was the limited adoption of social learning approaches by a few individuals rather than it being embraced by the entire organisation. Roux et al. (2011:4) concur that high staff turnover rates affect the ability to create inter-agency trust and working relationships and threaten continuity of initiatives, which is worsened by the general lack of skilled staff.

Inadequate funding was highlighted as a serious constraint. This was exacerbated by short project timeframes and delays in the allocation of resources. Other scholars, for example, Roux et al. (2011:4), noted that departments or agencies are sometimes informed about their budget allocations only halfway through the financial year, making it difficult to mobilise and spend resources, presenting challenges to planning and implementing project activities on time. When requirements and guidelines are rigid, coupled with delays in budget and resource allocation, activities to facilitate social learning become superficial and tokenistic. This situation calls for flexibility in project budgets and timeframes.

The limiting factors highlighted by the respondents are consistent with Alan and Wilson (2009:395) who found that short timeframes, project governance requirements and a focus on implementing organisation’s internal objectives constrained ecosystem restoration initiatives. Storbjörk (2010) observed that entrenched professional views and clashing organisational cultures hampered the reaching of consensus between human rights advocates, planners, decision makers and risk managers. When there is lack of consensus, it is difficult for stakeholders to work together for the successful implementation of programmes.

## Conclusion

The study assessed the prevalence of factors promoting social learning in climate change adaptation initiatives in South Africa. The findings provide some evidence of social learning in climate change adaptation projects in the country, with the majority of respondents indicating that personal social learning indicators were present. Mechanisms for improved conflict resolution were however less prevalent. The organisational and governance-related barriers to implementation present significant challenges. Key organisational barriers included short timeframes for implementing projects, inadequate financial resources, political interference, shortcomings in governance systems, and lack of knowledge and expertise in organisations. Efforts to promote social learning should be focused at both the organisational level as well as the individual level. There is a need for organisations to promote social learning by ensuring that their organisational environment and governance structures are conducive for their employees and the communities they work in, to embrace social learning.

While acknowledging that social learning is not a panacea to the climate change challenge, it is critical to stress that it is one of the essential ingredients in seeking to find long lasting solutions to the challenge through enhancing the adaptive capacity of communities. There is a need for people to have a shared understanding of the challenge and the recognition of the need and motivation to work together in tackling the challenge. A better understanding of the challenge and improved collaboration between individuals, organisations and communities are critical. Of significance is the need to incorporate different but complementary forms of knowledge (indigenous and modern). This will help contribute to the overall success of climate change adaptation initiatives. Optimal sustainable win-win solutions can result when stakeholders come together to collaborate on solutions with better understanding of the challenges and are equipped with appropriate technical skills.

## Footnotes

1 Some of the comments made by the respondents regarding the various factors are in quotation marks.
